# An Unusual Case of a Post-Traumatic Retained Periorbital Foreign Body in a Child

**DOI:** 10.7759/cureus.22478

**Published:** 2022-02-22

**Authors:** Mohmad Zulhisham, Ismail Shatriah

**Affiliations:** 1 Department of Ophthalmology and Visual Science, School of Medical Sciences, Universiti Sains Malaysia, Kota Bharu, MYS

**Keywords:** retained foreign body, eyelid foreign body, pediatric trauma, periorbital trauma, unwitnessed pediatric trauma, periorbital foreign body, foreign body, child

## Abstract

Trauma to the periorbital region is common in children. Foreign bodies in traumatic cases may be missed where history, examination, and investigation are inadequate. Here, we report the case of a four-year-old boy who presented with a small forehead scar and left upper lid swelling two months post-trauma. Imaging showed a retained foreign body, and a pen tip was successfully removed surgically from the lid. The child recovered well post-operatively. Complications such as orbital cellulitis may arise if the foreign body remained undetected.

## Introduction

The periorbital region is one of the most commonly affected regions in ocular trauma. In Malaysia, it was found that eyelid or eyebrow laceration is the second most common injury occurring in children [1]. Most surgical repair following ocular trauma in children involves the eyelids [1]. Embedded foreign bodies following trauma may be missed if history and examination are not done thoroughly. We report an unusual case of post-traumatic periorbital retained foreign body in a child.

## Case presentation

A four-year-old boy presented with non-resolving left upper lid swelling for two months. There was no pain or redness associated with the swelling. There was no decrease in vision. Further history revealed there was a history of fall from a table while playing, which was not witnessed by adults. The child suffered only a small forehead laceration that was sutured at a nearby primary health clinic previously. The child also had a left upper lid bruise that had resolved two weeks post-trauma; however, the swelling persisted. There were no other injuries.

**Figure 1:**
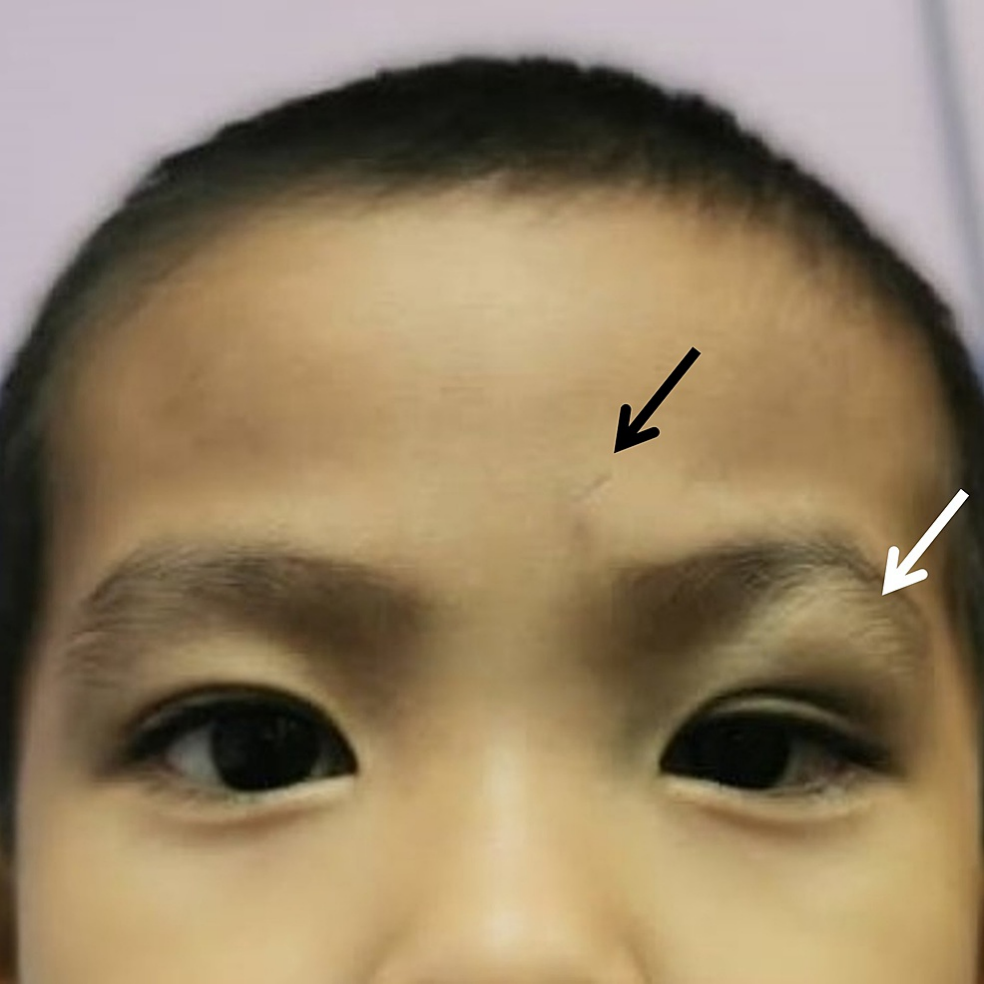
The child at presentation The black arrow indicates the forehead scar, and the white arrow indicates the left upper lid swelling.

The visual acuity was 6/9 (20/30) in both eyes. There was a hard and mobile swelling in the left upper lid measuring 2.5 cm x 2.5 cm. There was mild ptosis but no tenderness or erythema. A small scar was noted at the forehead about 1 cm superonasal to the eyebrow (Figure 1). No scar was seen at the lids. The bilateral extraocular movement was full. There was no relative afferent pupillary defect. Apart from the left upper lid, the anterior and posterior segment examinations were unremarkable.

**Figure 2:**
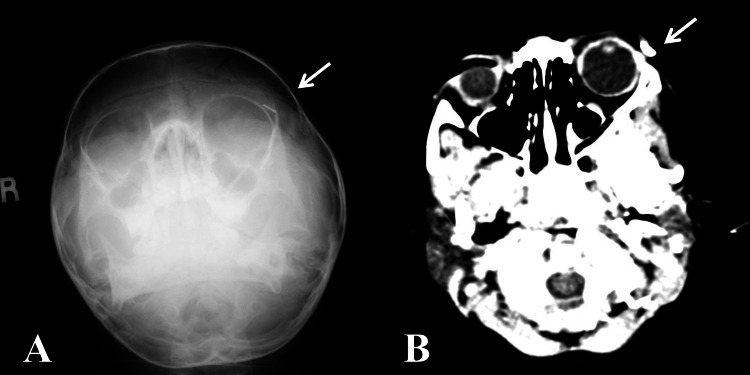
The foreign body (white arrow) in (A) Water's view skull x-ray and (B) CT

**Figure 3:**
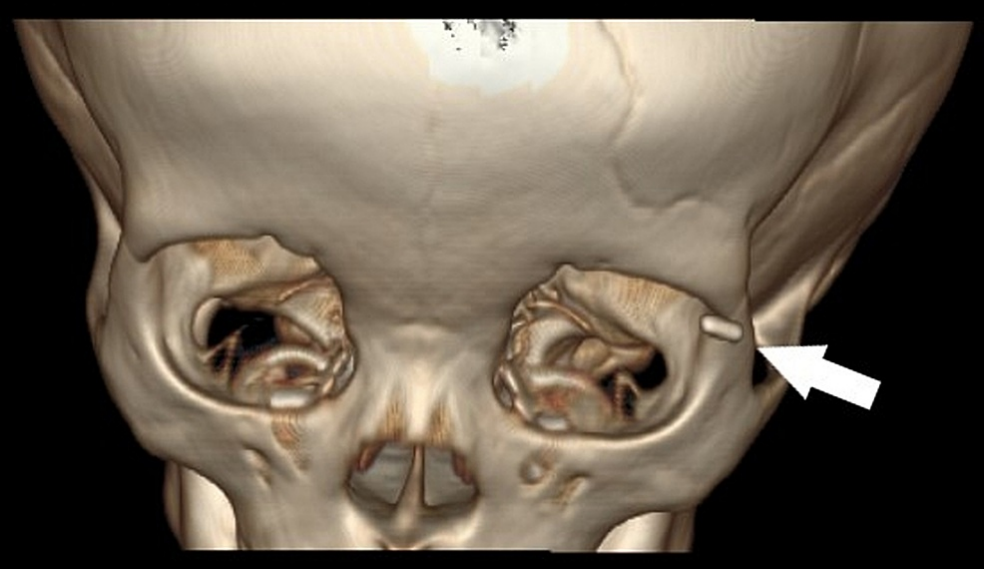
CT 3D reconstruction showing the exact location of the foreign body (white arrow)

A skull x-ray was done (Water’s view), which showed a short linear radiopaque foreign body in the left upper lid (Figure 2, Panel A). We further investigated with a contrast-enhanced computed tomography (CECT) orbit, which revealed a single linear dense material at the left periorbita with no focal collection (Figures 2B, 3). Other structures were unremarkable.

**Figure 4:**
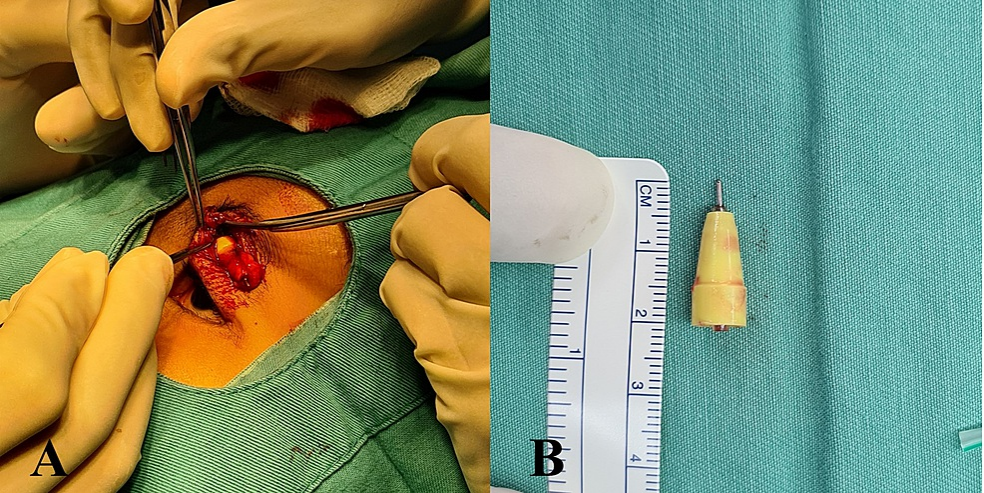
Surgical removal of the foreign body (A) and the foreign body, a pen tip, measuring 2.1 cm in length (B)

**Figure 5:**
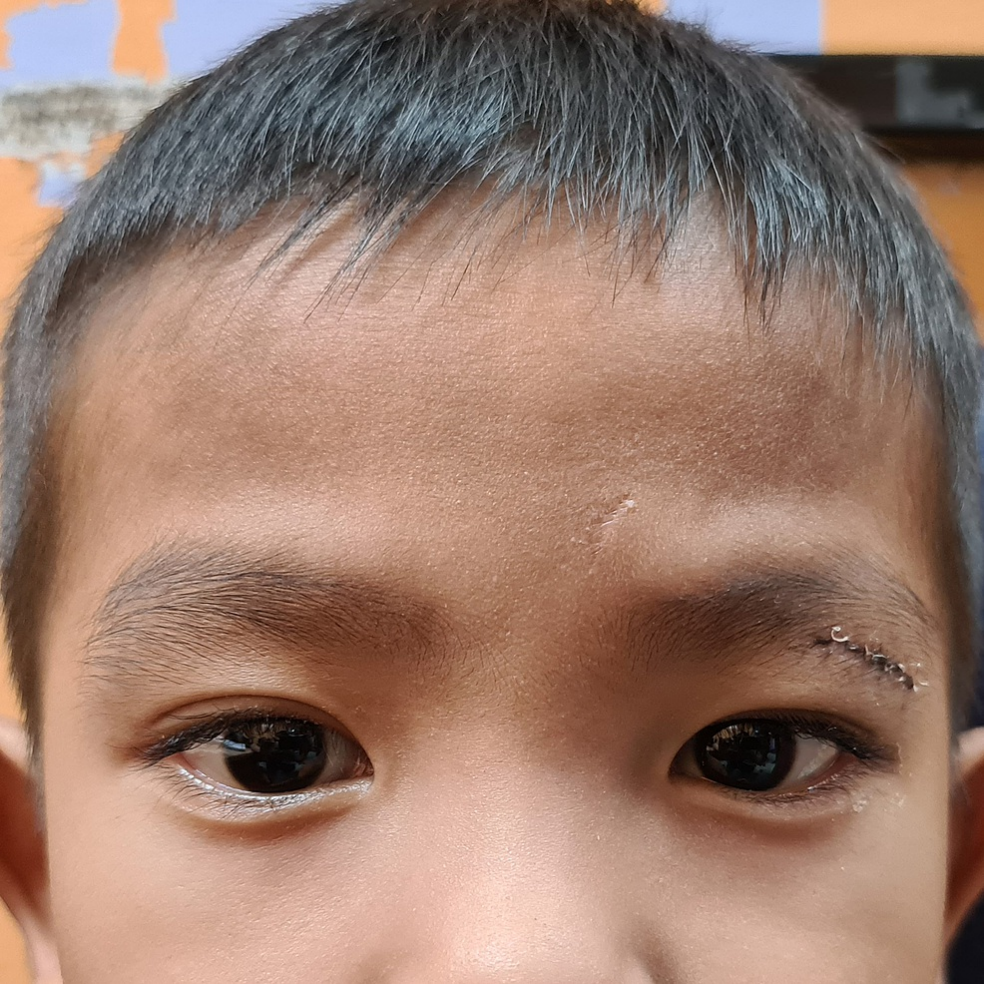
The child at post-operative review

Surgical exploration under general anesthesia revealed a pen tip measuring 2.1 cm in length, which was situated between the preseptal fat and orbicularis muscle (Figure 4). The pen tip and the surrounding granuloma were removed. Histopathology examination of the granulomatous tissue showed chronic inflammation changes consistent with the foreign body reaction. Post-operatively, the child was given a one-week course of oral amoxicillin/clavulanic acid 280 mg twice daily and ointment chloramphenicol three times daily over the wound. After two weeks, the wound healed well, and the swelling was completely resolved (Figure 5). The child was seen again at three months and discharged from our clinic.

## Discussion

Although not common, foreign bodies should be suspected in children following lid or periorbital laceration. A case series in Turkey noted seven out of 82 (8.5%) children had foreign bodies in their lid following ocular trauma [2]. Complications such as infection and fistula formation have been associated with retained foreign bodies [3]. This would potentially cause severe morbidity or mortality to the patient. As such, it is imperative to rule out foreign bodies in ocular trauma.

In our case, the foreign body was not detected by the primary care doctor at presentation due to several reasons. The initial injury and the location of the retained foreign body are unusual. We believed that it is possible that the child fell onto the pen that initially pierced the skin of his forehead and slid through the subcutaneous tissue until the pen tip reached the left upper lid. When the child removed the pen, the pen tip was separated and remained in the lid. The subsequent lid edema masked the presence of the pen tip making it difficult to detect visually during the examination. The trauma was unwitnessed by adults, which may have made history-taking incomplete and examination difficult as the child may have been irritable post-trauma. Furthermore, there is a possibility of the treating doctor’s attention only being paid to the treatment of the wound rather than to the proper diagnosis resulting in the foreign body not being detected [4].

In cases where it is difficult to obtain history or examination from the patient or relatives, imaging can be valuable to rule out the ocular foreign body. A readily available x-ray can be used to detect dense foreign bodies such as metals, stones, and glass materials, which are depicted as radiopaque on film. However, the use of x-rays on radiolucent material such as wood or plastic is limited as it is challenging to visualize those materials [5].

Computed tomography (CT) scan can be considered a gold standard for detecting foreign bodies as it is able to detect radiopaque and radiolucent materials [6]. However, surrounding tissue and artifact might be masked the appearance of a foreign body [6]. Therefore, care must be taken to interpret the imaging. Magnetic resonance imaging (MRI) is only rarely used for foreign body detection. This is due to its cost and availability [6]. Despite this, it may be helpful in detecting radiolucent materials, provided metallic foreign body has been ruled out by x-ray or CT [6].

A literature search was done looking at cases where there was prolonged retained periorbital foreign body following penetrating trauma. Only a few cases have been reported. Khanam et al. reported a case in a four-year-old boy who developed right orbital cellulitis five months post-trauma in which a plastic foreign body was embedded [7]. Another case was reported by Reddy in which a wooden foreign body was retained following a fall in an eight-year-old boy, which was not detected during initial examination [8]. In these two cases, the initial site of injury and the site of the retained foreign body were not far. One of the cases from a case series by Kıvanç et al. was nearly similar to our case, in which a three-year-old boy presented with a right upper lid mass of unknown origin, which turned out to be a metallic foreign body following a CT scan [2]. Compared to these three cases, our case was unusual due to the distance between the initial site of injury and the site of the foreign body. All these cases required the use of CT scan for foreign body detection and are summarized in Table 1.

**Table 1:** Summary of published cases on retained periorbital foreign body following penetrating trauma in pediatric patients

Author	Year	Age	Gender	Site of injury	Site of foreign body	Nature of the foreign body	Duration post-trauma	Complication
Reddy [8]	2013	8 years	Boy	Upper fornix conjunctiva	Right upper lid	Wood	10 days	Ptosis
Kıvanç et al. [2]	2019	3 years	Boy	Unknown	Right upper lid	Crotchet needle (metal)	1 week	Nil
Khanam et al. [7]	2021	4 years	Boy	Upper lid	Right medial wall of orbit	Plastic tube	5 months	Orbital cellulitis
Our case	2022	4 years	Boy	Forehead	Left upper lid	Pen tip (metal and plastic)	2 months	Nil

## Conclusions

A high index of suspicion of a foreign body should be maintained in cases of pediatric trauma of unknown etiology in which adult witness is absent. Thorough examination and investigations are required in such cases. Imaging such as x-ray and CT scans is valuable in foreign body detection. This case also highlights that a foreign body may be embedded further away from the initial wound, and care must be taken to assess properly during the initial presentation.

## References

[1] Min FCL, Qamaruddin F (2016). A West Malaysian study of pediatric ocular trauma. Int Eye Sci.

[2] Kıvanç SA, Ulusoy MO, Akova B, Atakan M (2019). Unusual foreign bodies in eyelids in childhood. CEOTI.

[3] Salazar-Ramos MS, Serna-Ojeda JC, Olvera-Morales O, Tovilla-Canales JL (2017). Periocular foreign body: two clinical cases with different management. Gac Med Mex.

[4] Zhou L, Li SY, Cui JP, Zhang ZY, Guan LN (2017). Analysis of missed diagnosis of orbital foreign bodies. Exp Ther Med.

[5] Tseng HJ, Hanna TN, Shuaib W, Aized M, Khosa F, Linnau KF (2015). Imaging foreign bodies: ingested, aspirated, and inserted. Ann Emerg Med.

[6] Voss JO, Maier C, Wüster J (2021). Imaging foreign bodies in head and neck trauma: a pictorial review. Insights Imaging.

[7] Khanam S, Agarwal A, Goel R (2021). Clinical presentation and management strategies in intraorbital foreign bodies. Case Rep Ophthalmol Med.

[8] Reddy SC (2013). Retained wooden foreign body in the orbit. Int J Ophthalmol.

